# Acupuncture treatment of restless legs syndrome: a randomized clinical controlled study protocol based on PET-CT and fMRI

**DOI:** 10.3389/fpsyt.2024.1481167

**Published:** 2024-12-24

**Authors:** Lin Tang, Na Zhao, Xiaolin Gao, Jinjin Li, Xintong Yu, Ruilong Liang, Chen Xie, Lutong Li, Qianqian Wang, Wenjia Yang

**Affiliations:** ^1^ Yueyang Hospital of Integrated Traditional Chinese and Western Medicine, Shanghai University of Traditional Chinese Medicine, Shanghai, China; ^2^ Department of Rehabilitative Medicine, Shanghai Fourth People’s Hospital Affiliated Tongji University, Shanghai, China; ^3^ Shanghai Research Institute of Acupuncture and Meridian, Shanghai University of Traditional Chinese Medicine, Shanghai, China

**Keywords:** acupuncture, restless legs syndrome, randomized controlled trial (RCT), PET-CT, fMRI, study protocol

## Abstract

**Introduction:**

Restless legs syndrome (RLS) is a sensorimotor disorder of the nervous system that is mainly characterized by nighttime leg discomfort and can be accompanied by significant anxiety, depression, and other mood disorders. RLS seriously affects the quality of life. Clinical studies have confirmed that acupuncture can alleviate the clinical symptoms of RLS. This randomized controlled trial (RCT) aims to investigate the efficacy of acupuncture in the treatment of RLS and further explore the central response mechanism of acupuncture in the treatment of RLS.

**Methods and analysis:**

In this RCT, a total of 124 eligible patients in Shanghai will be randomly assigned to one of the following two groups: treatment group (acupuncture) and control group (sham acupuncture). Treatment will be given three times per week for 4 consecutive weeks. The primary outcome is the International Restless Legs severity rating scale (IRLSS). The secondary outcomes are the RLS-Quality of Life (RLSQoL), the Insomnia Severity Index (ISI), Pittsburgh Sleep Quality Index (PSQI), the Hamilton Depression Scale (HAMD), and the Hamilton Anxiety Scale (HAMA). The objective evaluation tools will be polysomnography, positron emission tomography–computed tomography (PET-CT), and functional magnetic resonance imaging (fMRI) of the brain. All adverse effects will be assessed by the Treatment Emergent Symptom Scale. Outcomes will be evaluated at baseline (1 week before the first intervention), during the intervention (the second week of the intervention), after the intervention (at the end of the intervention), at 1-month follow-up, and at 3-month follow-up.

**Ethics and dissemination:**

The trial has been approved by the Ethics Committee of Yueyang Hospital of Integrated Traditional Chinese and Western Medicine (no. 2022-061). Written informed consent will be obtained from all participants. The results of this study will be published in peer-reviewed journals or presented at academic conferences.

**Clinical trial registration:**

https://www.chictr.org.cn/, identifier ChiCTR2000037287.

## Introduction

Restless legs syndrome (RLS) is a neurosensory motor disorder (1) with nighttime leg discomfort as the main feature. It can be accompanied by obvious emotional disorders such as anxiety and depression, seriously affecting the quality of life. Epidemiological surveys have shown that the prevalence of adult RLS is different in different countries and regions. The pooled estimated RLS prevalence by region is the highest in South America (16%) and Europe (16%), followed by Africa (12%), North America (9%), and Asia (6%) (2).

RLS can occur at any age, but with age, the prevalence increases year by year. Based on a population-based survey, the prevalence of RLS is 3% between 18 and 29 years, 10% between 30 and 79 years, and 19% in individuals older than 80 years (3). The prevalence is higher in women than in men (2, 4). RLS has both sensory and motor symptoms. The typical clinical symptom group mainly appears at night, which seriously interferes with sleep, causing difficulty falling asleep and increasing the number of night awakenings, and then fatigue, memory loss, depression, and blood pressure fluctuations, which affects the quality of life. Various functions during the day are also negatively affected (5–7). A systematic retrospective analysis of RLS (8) has shown that the prevalence of patients with anxiety and depression increases significantly. Our previous research has also shown the impact of RLS on physical and mental exhaustion during the day. Patients with RLS have obvious mood disorders such as anxiety and depression. It has been further confirmed in the Chinese population that RLS can significantly increase the risk of mood disorders (9), and an increasing number of studies have shown that RLS can increase the risk of cardiovascular disease (10, 11). Currently, Chinese scholars are also conducting large cohort studies on the potential association between RLS and cardiovascular diseases, which will provide more evidence for the intervention of related diseases in the future (12). RLS is not uncommon in clinical practice, and it is particularly worth noting in the context of Parkinson’s disease and RLS, where the prevalence of nighttime problems including probable rapid eye movement sleep behavior disorder and RLS is 9.5%, significantly higher than the prevalence of any other combination of sleep disorders (13). Because patients have low awareness of the disease, only a minority of patients with RLS go to hospital for treatment. The diagnosis is mainly based on clinical symptoms and lacks specificity. Therefore, the current diagnosis rate of the disease is relatively low. RLS is often misdiagnosed as arthritis, peripheral neuropathy, insufficient blood supply to the lower limbs, and neurosis, resulting in delayed diagnosis and treatment (14, 15). At present, the drugs used to treat RLS mainly include dopaminergic drugs, antiepileptic drugs, benzodiazepines, and opioids. However, long-term use of benzodiazepines can easily cause drug dependence, while long-term use of antidepressant or anxiolytic drugs can cause many adverse reactions. As a new generation of non-ergot dopamine receptor agonists, pramipexole not only has high specificity for dopamine receptors but also has complete intrinsic activity. It is approved for the treatment of RLS (16). However, the rebound phenomenon is the main complication of long-term use of dopaminergic drugs (17). According to research reports, the incidence of the rebound phenomenon is 27.1% in RLS patients treated with levodopa and 6.0% in RLS patients treated with pramipexole (18). Due to the low rate of correct diagnosis and treatment of RLS and the lack of long-term effective drug treatment, there is an urgent need for a treatment method with definite curative effects and few side effects.

Chinese medicine has had a clear understanding of RLS. “The patient has trouble sleeping at night and has a feeling of soreness and hotness in the legs. This kind of feeling continues if the patient does not fall asleep when he lies on the bed. In addition, the leg muscles could have spasms, leading to frequent leg movements from left to right or from right to left, until the patient falls asleep from exhaustion.” This is Xue Ji’s description of restless legs in 1529 (19). Traditional Chinese medicine believes that the liver governs the tendons, and the spleen governs the flesh, so the disease is most closely related to the liver and the spleen (20). The liver controls the tendons and veins of the whole body, and the spleen controls the muscles of the whole body. If the two are abnormal, the bones and muscles are not nourished, which causes restless legs (21). Long-term clinical practice has proven that traditional Chinese medicine has a good curative effect in the treatment of RLS, and Chinese medicine has been widely used in the clinical treatment of RLS. Acupuncture plays an important role in clinical treatment. With the continuous deepening of research in recent years, the unique advantages of acupuncture and moxibustion in the prevention and treatment of RLS have gradually emerged and attracted widespread attention. Clinical trials have also confirmed that acupuncture has significant efficacy in the treatment of RLS, is easy to operate, has no toxic side effects, and is easily accepted by patients (22).

The pathogenesis of RLS is not yet clear. It may be closely related to the abnormality of the dopamine (DA) pathway. The current research on RLS confirms the presence of abnormalities in the DA pathway of the spinal cord transmission system (23, 24), the DA pathway in the limbic system of the midbrain cortex (25), and the DA pathway in the substantia nigra striatum (26). Our previous experimental results have also confirmed that the cortex–striatum–thalamus dopaminergic system plays an essential role in the pathogenesis of RLS (27). With the rapid development of neuroimaging technology, there is an increasing number of imaging studies to explore the central nervous system function and metabolism, which provides us with a lot of useful information for an in-depth understanding of the pathogenesis of RLS. Positron emission tomography–computed tomography (PET-CT) has confirmed that the DA pathway is abnormal in RLS, which involves not only the substantia nigra striatum pathway but also the midbrain limbic pathway. Hence, the DA pathways in the substantia nigra striatum and the midbrain limbus are abnormal, which further causes the sensorimotor network and limbic network to dysfunction, resulting in RLS. Functional magnetic resonance imaging (fMRI) studies have shown changes in the neural activity and functional connection of the sensorimotor network and the limbic network. RLS patients have higher local consistency (ReHo) in the striatum, thalamus, and limbic system, and emotional processing, motor regulation, and abnormal cognitive regulation suggest that the cortex–striatum–thalamus–cortical circuit is involved in the pathogenesis of RLS (28). Epidemiological data have shown that due to the lack of knowledge about RLS by clinicians and the crossover of RLS with other diseases in clinical symptoms, only 6.2% of patients are correctly diagnosed in the end (14, 15). Functional imaging technologies such as PET-CT and fMRI help understand the clinical complexity, intermittentness, and diversity of this disease, can improve the specificity and sensitivity of clinical diagnosis, and may ultimately help clinical treatment and prognosis.

The incidence of RLS gradually increases with age, and the rate of clinically correct diagnosis and treatment is low. At present, there is still a lack of long-term effective therapeutic drugs. Clinical studies have confirmed that acupuncture can relieve the clinical symptoms of RLS and is easy to operate without toxic side effects.

However, high-quality randomized clinical trials (RCTs) are needed to evaluate the clinical efficacy of acupuncture for RLS. This study will adopt a patient-blinded, randomized, controlled clinical trial design, and enroll patients with RLS who meet the diagnostic criteria and who are receiving acupuncture for the first time. We will use subjective scales, polysomnography, and functional imaging techniques to comprehensively evaluate the clinical efficacy. The results will help to demonstrate whether acupuncture is an effective and safe therapy for improving the clinical symptoms of RLS.

Current studies that have incorporated acupuncture into RLS treatment protocols have shown promising results in symptom relief. However, these studies lack the depth of neuroimaging data that could elucidate the underlying mechanisms of how acupuncture might influence the neural pathways associated with RLS (29, 30). Thus, we will further explore the central mechanism of acupuncture for treating RLS by PET-CT and fMRI. Based on current neuroimaging studies on RLS (26, 31–33), we expect that acupuncture treatment could regulate the dopamine transporter (DAT) to enhance dopamine activity in the brain, and by altering the expression of certain genes and proteins, it may increase the availability of dopamine, thereby intervening in RLS. Additionally, changes in the functional connectivity and activity within the sensorimotor and limbic networks following acupuncture treatment could be identified. Thus, the integration of PET-CT and fMRI data will provide a comprehensive understanding of how acupuncture influences the neural mechanisms of RLS.

## Methods

### Study design

This is a randomized, single-blind, placebo-controlled clinical trial, aimed at evaluating the efficacy and safety of acupuncture for RLS patients and comparing the effects between acupuncture care and sham acupuncture care. The trial will be performed at Yueyang Hospital of Integrated Traditional Chinese and Western Medicine of Shanghai University of Traditional Chinese Medicine. We will recruit 124 patients who meet the inclusion criteria and randomly assign them to either the acupuncture or sham acupuncture groups. Basic information and baseline data will be collected in the first week after enrollment, after which the participants will enter a 1-month observation period in this trial. All treatments will be given three times a week (every other day) for 4 weeks.

**Figure 1:**
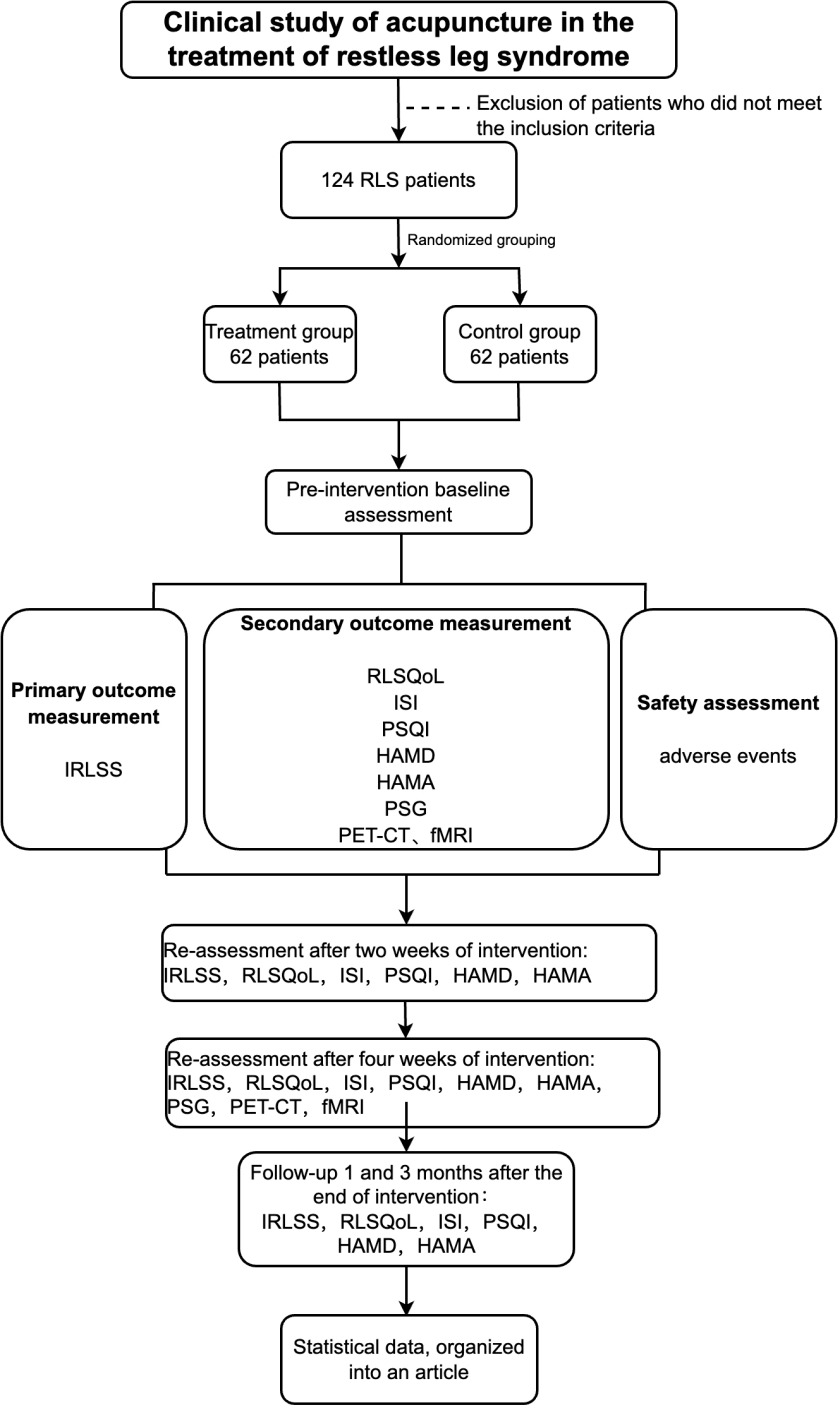
Flow chart.

The participants will be assessed at the following time points: the baseline (1 week before the treatment), the middle of the treatment (2 weeks after the treatment start), the end of the treatment (4 weeks after the treatment start), and the follow-up (1 month and 3 months after the treatment end). We will use subjective scales and polysomnography combined with functional imaging technology for comprehensive evaluation of clinical efficacy. We will use the International Restless Legs severity rating scale (IRLSS) to evaluate the symptoms of restless legs and the RLS Quality of Life (RLSQoL) to evaluate the patients’ quality of life. Insomnia Severity Index (ISI), and the Pittsburgh Sleep Quality Index (PSQI) will be used to evaluate the patients’ sleep status, and the Hamilton Depression Scale (HAMD) and Hamilton Anxiety Scale (HAMA) will be used to evaluate the mood of the patients. We will use polysomnography (PSG) to analyze the objective sleep quality of the patients, and fMRI and PET-CT will be used to monitor brain function. The detailed trial process is shown in **Figure 1** and **Table 1**. This study will follow the Consolidated Standards of Reporting Trials (CONSORT) (34) and the Standards for Reporting Interventions in Clinical Trials of Acupuncture (STRICTA) (35) guidelines for designing and reporting controlled trials.

**Table 1:** Trial process chart.

	Baseline		Treatment phase				Follow-up phase	
	Week	Week	Week	Week	Week	Week	Month	Month
	-1	0	1	2	3	4	1	3
Patients								
Telephone reservation	×							
Enrollment	×							
Signed informed consent		×						
Clinical interview		×						
Physical examination		×						
Laboratory test		×						
Randomization		×						
Groups								
Acupuncture group			12 treatments					
Control group			12 treatments					
Primary outcomes								
**IRLSS**		×		×		×	×	×
Secondary outcomes								
RLSQoL		×		×		×	×	×
ISI		×		×		×	×	×
PSQI		×		×		×	×	×
HAMD		×		×		×	×	×
HAMA		×		×		×	×	×
PSG		×				×		
PET-CT		×				×		
fMRI		×				×		
TESS			×	×	×	×		
Drug dose record		×	×	×	×	×	×	×
Patient’s compliance			×	×	×	×	×	×

IRLSS, the International Restless Legs severity rating scale; RLSQoL, the RLS Quality of Life; ISI, the Insomnia Severity Index; PSQI, Pittsburgh Sleep Quality Index; HAMD, Hamilton Rating Scale for Depression; HAMA, Hamilton Anxiety Sale; PSG, polysomnography; PET-CT, Positron Emission Tomography-Computed Tomography; fMRI, functional magnetic resonance imaging; TESS, Treatment Emergent Symptom Scale.

#### Participants

This study will include 124 RLS patients. To ensure the precision of the results, we developed the following eligibility criteria.

#### Inclusion criteria

1. Male or female participants aged 18–75 years;
2. Patients with primary RLS meeting the 2014 International RLS Research Group diagnostic criteria (1);
3. No abnormalities in physical examination of the nervous system;
4. Participants who have not received acupuncture treatment before;
5. Participants who voluntarily agree with the investigation and sign a written informed consent form for the clinical trial.

#### Exclusion criteria

Participants who report any of the following conditions will be excluded:

1. Secondary RLS caused by iron-deficiency anemia, rheumatoid arthritis, Parkinson’s disease, peripheral neuropathy, and other diseases;
2. Participants with other organic diseases, such as cardiovascular, liver, kidney, lung, and other serious primary diseases, or severe mental symptoms;
3. Pregnant and lactating women;
4. Participants who took drugs that affect sleep and periodic limb movement index changes (such as tricyclic antidepressants, sleeping pills, and dopamine drugs) within 2 weeks before the examination;
5. Participants who refuse acupuncture, fMRI, and PET-CT;
6. Participants who have participated in other clinical studies 3 months before enrollment or are participating in other clinical studies.

#### Recruitment

This study will include 124 participants with RLS. We plan to recruit participants through hospital-based advertisements from outpatient clinics and from official websites. We will also recruit participants through newspapers, social media, and advertising. The recruitment location will be restricted to Yueyang Hospital of Integrated Traditional Chinese and Western Medicine affiliated with the Shanghai University of Traditional Chinese Medicine. If RLS patients are interested in participating in the trial, they can complete the phone screening first and then will be asked for face-to-face screening in the hospital, where they need to fill in some forms with guidance from doctors with professional training. Once the participants meet the inclusion criteria, they will be asked to sign the written informed consent form before the intervention begins.

### Interventions

All acupuncturists are licensed doctors with 10–15 years of experience in acupuncture treatment, and they will participate in the training before the intervention to ensure the standard real and sham acupuncture operations. The participants in the treatment group and the control group will receive acupuncture and sham acupuncture treatments, respectively. The participants in these two groups will receive 12 sessions of different treatments, three times a week for 4 weeks. After skin cleansing, the patients will lie on their back and receive acupuncture or sham acupuncture treatment. Each treatment will last for 30 min. The temperature of the treatment room will not be lower than 26°C.

#### Treatment group

**Figure 2:**
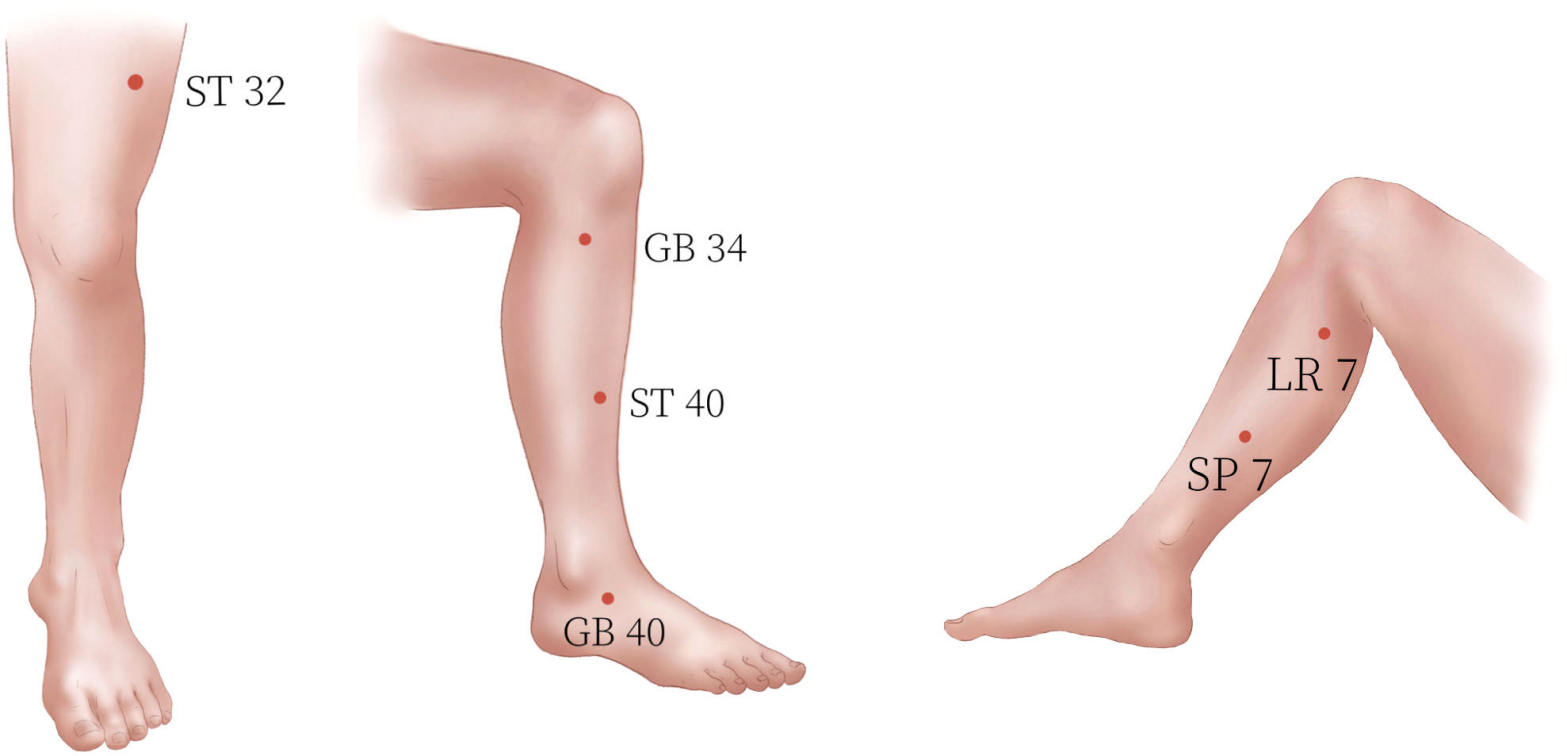
Locations of the acupoints.

**Figure 3:**
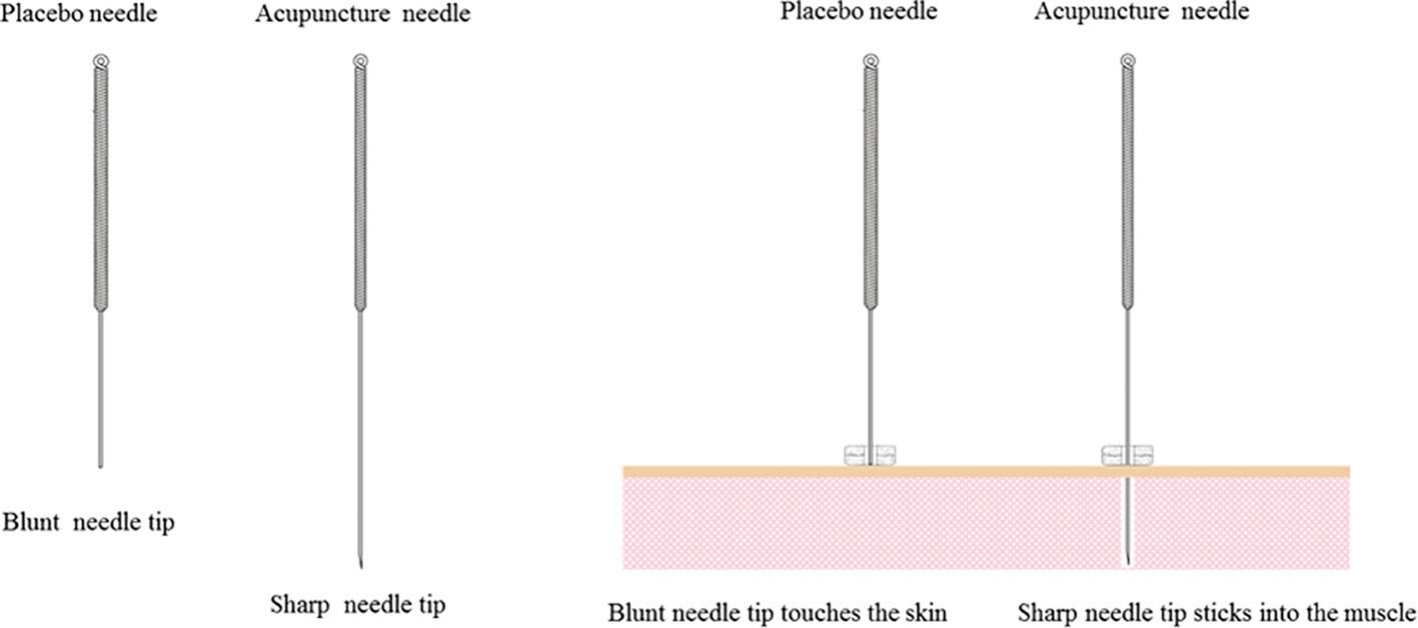
Huatuo acupuncture needle and placebo needle.

The participants in the treatment group will receive acupuncture treatment. They will be placed in the supine position. We have selected the following six acupoints for this study: Yanglingquan (GB 34), Xiguan (LR 7), Qiuxu (GB 40), Futu (ST 32), Fenglong (ST 40), and Lougu (SP 7). The reasons for choosing these acupoints are the basic theory of TCM and our clinical experience. The location of each acupoint is shown in **Table 2** and **Figure 2**. Huatuo disposable needles (Suzhou Medical Appliance, Jiangsu, China) (shown in **Figure 3**) with a length of 40 mm and a diameter of 0.25 mm, or a length of 50 mm and a diameter of 0.30 mm, will be used. The acupuncturist will stimulate points on both sides of the body. After piercing, a thrusting and twirling motion of the needle will be performed to induce the sensation known as ‘De qi,’ and the needle will then be left in place for 30 min. De qi means that after the needle has penetrated the acupoint to a certain depth, the needle is thrust and twirled to cause the acupoint to obtain the induction of meridian qi. The subjects will have a self-conscious reaction such as soreness, heaviness, or distention. This is the key process in acupuncture treatment (36). After the acupuncture is finished, the needles will be quickly removed, and the needle holes will be pressed to avoid bleeding or subcutaneous hematoma. The treatment will be performed three times a week for a total of 4 weeks.

**Table 2:** Location for each acupoint.

Acupoint	Location
Yanglingquan (GB 34)	In the depression anterior and inferior to the head of the fibula.
Xiguan (LR 7)	Posterior and inferior to the medial condyle of the tibia, in the upper portion of the medial head of m. gastrocnemius, 1 cun posterior to Yinlingquan (SP 9).
Qiuxu (GB 40)	Anterior and inferior to the external malleolus, in the depression on the lateral side of the tendon of m. extensor digitorum longus
Futu (ST32)	On the line connection the anterior superior iliac spine and lateral border of the patella, 6 cun above the laterosuperior border of the patella.
Fenglong (ST 40)	8 cun superior to the tip of the external malleolus, lateral to Tiaokou (ST 38) about two finger-breadth lateral to the anterior border of the tibia.
Lougu (SP 7)	6 cun from the tip of the medial malleolus, on the line connecting the tip of the medial malleolus and Yinglingquan (SP 9), posterior to the medial border of the tibia

#### Control group

The participants in the control group will receive sham acupuncture treatment at the same acupoints as the treatment group. Sham acupuncture will be applied with Huatuo placebo needles (Suzhou Medical Appliance, Jiangsu, China; shown in **Figure 3**), which have been successfully used in a previous study (37). The patients will experience a tingling sensation when the tip of the blunt needle touches the skin, but there will be no real needle inserted into the skin. After 30 min of treatment, the acupuncturists will inform the patients that they will be removing the needle and using sterile dry cotton balls to press the acupoints. The treatment will be performed three times a week for a total of 4 weeks.

### Outcome measures

#### Primary outcome measures

1. IRLSS (38): This scale quantitatively scores the severity of the patient’s RLS, with 10 points, 20 points, and 30 points used as the cutoff points to divide the severity of RLS into mild, moderate, severe, and extremely severe, respectively.

#### Secondary outcome measures

1. RLSQoL (39): This 18-item scale will be used to assess the quality of life of the patients with RLS in the past month. The lower score indicates poorer quality of life.
2. ISI (40): The ISI is mainly used to evaluate the nature and severity of insomnia and the impact on the patient’s daytime function. It consists of seven items. The total score of the seven items is the total ISI score. The higher the degree of insomnia, the higher the score. A total score of 8–14 points has subclinical significance. For insomnia, a total score > 14 represents clinically significant insomnia.
3. PSQI (41): It is composed of 19 self-assessed items that can be combined into seven factors (sleep quality, time to fall asleep, sleep time, sleep efficiency, sleep disorders, hypnotics, and daytime function), and each factor is scored on a scale of 0-3. The cumulative score of each factor component is the total score. Individuals with a score ≥ 8 are considered to have poor sleep quality.
4. HAMD (42): The HAMD scale was compiled by Hamilton in 1960. In this study, the 17-item HAMD scale will be used. The scale is composed of five factors, namely anxiety/somatization, weight, cognitive impairment, retardation, and sleep disturbance, with a total of 17 items. A total score< 7 indicates normal state; a total score of 7–17 indicates mild depression; a total score of 17–24 indicates moderate depression; and a total score > 24 indicates severe depression. The HAMD scale has good reliability and validity. Clinically, it has become the most common scale to evaluate the severity of depressive symptoms.
5. HAMA (43): The HAMA scale was compiled by Hamilton in 1959 with 14 entries. A total score< 7 indicates normal state; a total score of 7–14 indicates mild anxiety; a total score of 17–24 indicates moderate anxiety; and a total score > 24 indicates severe anxiety. The HAMA scale has good reliability and validity.
6. PSG: PSG (EEG-1200C Nihon Kohden, Japan) will be used to record sleep all night, and the monitoring time will be at least 8 h. The recorded sleep physiological parameters will include total bedtime, total sleep time, number of awakenings, sleep latency, rapid eye movement sleep latency, number of sleep staging transitions, number of awakenings, microwake index, wake time after falling asleep, proportion of sleep in each period, periodic limb movements during wake (PLMW) index, periodic limb movements during sleep (PLMS) index, and PLMS-arousal index. After the monitoring, a professional sleep technician will be engaged for manual analysis based on the American Sleep Medicine Association’s sleep and related event interpretation standards (44).
7. PET-CT: Patients will undergo positron emission tomography/computerized tomography examination using (11)C-2b-carbomethoxy-3b-(4-fluorophenyl) tropane ( (11)C-CFT). During the (11)C-CFT PET imaging, which will be undertaken 60 min following the intravenous injection of CFT (350–400 MBq), scanning data will be collected for 20 min and then reconstructed using the ordered subsets expectation maximization (OSEM) method. All PET data will be captured in three-dimensional (3D) mode. Subsequently, the (11)C-CFT PET data will be reconstructed using SPM5 software (Statistical Parametric Mapping; Wellcome Department of Imaging Neuroscience, Institute of Neurology, London, UK) implemented in Matlab7.4.0 (MathWorks Inc., Sherborn, MA, USA). This analysis will be used to investigate changes in the dopamine transporter within the patient’s brain.
8. fMRI: Conventional structural MRI scans of the subjects, including T1-weighted imaging (T1WI), T2-weighted imaging (T2WI), diffusion-weighted imaging (DWI), and T2 fluid-attenuated inversion recovery (T2FLAIR) sequences, will be used for screening individuals with organic brain diseases. Following this, a resting-state fMRI scan will be conducted with the following parameters: duration = 450 s, TR=2000 ms, TE=30 ms, matrix size = 64 × 64, field of view (FOV) = 230 × 230 mm^2^, slice thickness = 4 mm, and slice gap = 0.8 mm. Once the MRI scan is complete, the subject’s data will promptly be saved in Digital Imaging and Communications in Medicine (DICOM) format and stored on a mobile storage device. To process the resting-state fMRI data, we will utilize the Resting-State fMRI Data Processing Assistant (DPARSFA) V2.1 (http://www.restfmri.net) within the MATLAB 7.8 (R2009) platform.

### Safety assessments

We will count adverse events during the trial. Adverse events will be defined as any adverse medical reactions that have occurred from the time the subject signed the informed consent form to the time of the last follow-up, whether or not there is a causal relationship with the study treatment. All adverse effects will be assessed by the Treatment Emergent Symptom Scale. We will record the time point, severity, measures taken, whether it is related to treatment, and prognosis of adverse events (45). Investigators will assess the possible relationship of adverse events to this study and provide appropriate treatment and guidance.

### Sample size

The sample calculation is based on changes in the primary outcome of this trial, which is the IRLSS score, indicating the severity of the patient’s RLS. In our previous study, the IRLSS score of the placebo group was 20.02, and the standard deviation σ was 6.44. This trial adopts an equal difference (1:1) design plan, divided into the treatment group and the control group. We hypothesize that the acupuncture in our study could reduce the IRLSS score by 4 points compared with the placebo acupuncture, and σ=7.26. According to the needs of this study, we set α=0.05 and 1−β=0.90, according to the formula:


n1=n2=2(Zα2+Zβ)2×σ2(μ2−μ1)2

We calculated n = 52 as the sample size of each group. Considering the loss factor (according to a 15% loss rate), the number of samples in each group will be 62 cases, with a total of 124 cases across the four subgroups.

### Randomization and blinding

Statisticians will use SPSS V.26.0 to generate a random-number list, and the patients will be randomized into the treatment group or the control group in a 1:1 ratio according to the order in which they have signed the informed consent. The random number and treatment plan will be printed out and put into sealed opaque envelopes. The envelopes will be numbered, and the enrolled patients will be randomly grouped by matching the numbers on the envelopes in order of inclusion. During the trial, the generation of the random number list, patient recruitment, acupuncture treatment, outcome measurement, and data analysis are all conducted by specialized personnel who do not exchange information with each other. Only the acupuncturist will know the allocation. The patients and other researchers will be blinded to assignments.

### Data collection and management

To ensure standardization, researchers will be trained uniformly before the trial, including questionnaire survey, clinical treatment, indicator detection, data collection, and management. The questionnaire survey will be arranged for researchers to complete, and the researchers must fully understand the contents of the questionnaire. During the experiment, researchers should carefully implement informed consent and patiently explain the study to the subjects so that they can fully understand and cooperate with the study. Questionnaire investigators will perform patient interpretation and truthfully record the patient’s questionnaire situation. Clinical treatment will be carried out by fixed doctors, and the operation methods of acupuncture and sham acupuncture will be unified. In the process of treatment, clinicians will standardize operations, try to avoid unnecessary communication with the patients, and not inform the patients of the treatment plan. fMRI and PET-CT scans will be performed by stationary personnel using the same scanner and analyzed.

Data will be collected at the baseline (1 week before the first intervention), during the intervention (the second week of the intervention), after the intervention (at the end of the intervention), at 1-month follow-up, and at 3-month follow-up. We will collect data at baseline and after the intervention, including the following assessments: the IRLSS for evaluating the severity of RLS, the RLSQoL for reflecting the quality of life of patients with RLS, the ISI for reflecting the severity of insomnia, the PSQI for assessing sleep quality, the HAMD for reflecting the depressive mood of the subjects, the HAMA for reflecting the anxiety mood of the subjects, and the PSG for analyzing the sleep process and sleep structure throughout the night. Additionally, we will conduct PET-CT and fMRI scans of the brain.

The data we will collect during the second week of the intervention, at 1-month follow-up, and at 3-month follow-up will include IRLSS, RLSQoL, ISI, PSQI, HAMD, and HAMA. Data entry and management will be completed by the data manager, and data records will be timely, accurate, complete, and standardized. The data manager will use software for data entry and management. After the data manager confirms that the database is established correctly, the main researcher and statistical analyst will lock the data. The research process will be kept strictly confidential, and the locked data file will not be altered.

### Statistical analysis

Data analyses will be performed with the use of the statistical software SPSS V.26.0 (IBM SPSS Statistics, IBM Corp, Somers, NY, USA). Statistical analysis will be conducted by two analytical researchers independent of the experiment, following the principle of intention-to-treat (ITT) analysis. In this study, we plan to adopt a comprehensive strategy for handling missing data. Firstly, we will identify the mechanisms of data missingness, distinguishing between Missing Completely At Random (MCAR), Missing At Random (MAR), and Missing Not At Random (MNAR). We took preventive measures against data loss through carefully designed study procedures and data collection protocols, resorting to imputation techniques when necessary to maintain dataset integrity. Additionally, we plan to conduct sensitivity analyses to assess the impact of different missing data assumptions on study outcomes. Specifically, we will utilize Latent Growth Modeling (LGM) to estimate missing data under MAR and MNAR conditions, comparing results from different models to examine the robustness of our conclusions. Through these methods, we aim to ensure the reliability and validity of study results even in the presence of incomplete data. For the measurement data that conform to the normal distribution and homogeneous variance, the mean ± standard deviation (SD) will be used to describe the discrete tendency and central tendency. The comparison between the two groups will be performed by the independent-samples *t* test. Comparisons at multiple time points will be made using repeated-measure analysis of variance. Measurement data that do not conform to the normal distribution will be described by median and interquartile range. Comparison between the two groups will be conducted using the Mann–Whitney *U* rank-sum test. The comparison of multiple time points will be based on the generalized estimating equation (GEE). Enumeration data will be expressed by frequency (n) and constituent ratio (%). Comparison between the groups will be performed by the X² test. When the data do not meet the conditions of the X² test, the Fisher’s exact probability method will be used. In this study, all statistical tests will be performed two-sided, and P<0.05 will indicate that the difference is statistically significant.

## Discussion

RLS is characterized by nighttime leg discomfort, which can be accompanied by apparent emotional disorders such as anxiety and depression, seriously affecting the quality of life. The diagnosis of RLS mainly relies on clinical symptoms and lacks specificity. Due to the low rate of correct diagnosis and treatment of RLS and the lack of long-term effective drug treatment, there is an urgent need for a treatment method with definite curative effects and few side effects. Because acupuncture has the characteristics of safety, efficacy, and few side effects, it may provide an effective method for the treatment of RLS.

Previous clinical studies have mainly focused on the clinical efficacy of acupuncture and moxibustion in the treatment of RLS, and the indicators were relatively single. Moreover, few authors have focused on the effects of acupuncture on RLS-related emotions, and there has been no mechanistic exploration of the acupuncture treatment for RLS. Our trial was designed to provide a rigorously designed study to investigate the effects of acupuncture in patients with RLS. We will use subjective and objective indicators to evaluate the clinical efficacy of acupuncture in the treatment of RLS. For the first time, we will further explore the central response mechanism of the acupuncture treatment of RLS by functional imaging technology.

For patients in the acupuncture group, we decided to use acupuncture at Yanglingquan (GB 34), Xiguan (LR 7), Qiuxu (GB 40), Futu (ST 32), Fenglong (ST 40), and Lougu (SP 7). According to traditional Chinese medicine theory, the liver governs the tendons, and the spleen governs the flesh. Since RLS is mainly associated with discomfort in the lower extremities, traditional Chinese medicine believes that the onset of restless legs is most closely related to the liver and spleen. In traditional Chinese medicine theory, there is a close relationship between the liver meridian and the gallbladder meridian in terms of both physiology and pathology. The acupoints of the gallbladder meridian can be used to treat liver diseases. We have chosen Xiguan (LR 7) for the liver meridian and Yanglingquan (GB 34) and Qiuxu (GB 40) for the gallbladder meridian, as they can treat diseases that predominantly involve the liver. Similarly, there is a close relationship between the spleen meridian and the stomach meridian in terms of physiology and pathology, and the acupoints of the stomach meridian can be used to treat diseases of the spleen. We chose Lougu (SP 7) of the spleen meridian and Futu (ST 32) and Fenglong (ST 40) of the stomach meridian to treat diseases dominated by the spleen. In the technique of acupuncture, we adopted articular needling combined with muscle needling in “Neijing.” Articular needling refers to inserting the needle into the joints of the limbs to treat diseases of joint tendons (26). Yanglingquan (GB 34), Xiguan (LR 7), and Qiuxu (GB 40) are found at the knee joint and ankle joint, so we will apply articular needling at these three points. Muscle needling is a method of penetrating needles deep into muscles to treat muscle diseases (26). Futu (ST 32), Fenglong (ST 40), and Lougu (SP 7) are located in areas with lower limb muscles; therefore, we will use muscle needling at these three points. Therefore, we selected these six acupoints and adopted articular needling combined with muscle needling of Neijing to treat restless legs, which can effectively relieve the discomfort symptoms of lower limbs in patients with RLS. In our previous study, we confirmed the clinical efficacy of articular needling combined with muscle needling in the treatment of RLS. In actual acupuncture clinical trials, it is challenging to blind the practitioners, as they can often discern whether they are performing acupuncture or sham acupuncture. Therefore, we have chosen a single-blind design for this research study protocol. However, single-blind trials also have certain limitations, such as the method of sham acupuncture, which may be detected by subjects with experience in acupuncture. Additionally, due to cultural and cognitive differences, populations in Asian countries like China have a higher acceptance of acupuncture and a deeper understanding and expectation of it, which may lead to easier unblinding during the study or influence the trial results due to patients’ subjective expectations (46). Taking these issues into account, we have set the inclusion criterion that patients have never received acupuncture treatment before.

For the evaluation of the clinical efficacy of acupuncture for RLS, we will combine subjective and objective indicators. We will use IRLSS to evaluate the subjective feelings of the patients and use PSG to monitor their PLMS. The International Restless Legs Study Group (IRLSSG) has developed the IRLSS (International Restless Legs Syndrome Severity Scale), which is a widely used measure for the evaluation of RLS severity (47). The PSG provides a direct measure of PLMS, which has been defined as a series of at least four leg movements each lasting 0.5–10 s with 5–90 s from onset to onset of consecutive movements (48). They characteristically occur in the sleep of RLS patients, and in one large series occurred at a high rate (>5/hour) in 80% of RLS patients. These leg movements are sufficiently distinct and common among RLS patients to be considered a motor sign of RLS (49). Documenting sleep status and particularly the PLMS may provide a very useful assessment of RLS morbidity (50). In 2006, the IRLSSG developed and published the scoring criteria for periodic leg movements (PLMs) (48). The IRLSSG developed and published new consensus standards for scoring PLMs in 2016, which are now the official standards for the World Association of Sleep Medicine and the World Sleep Society (51). Nearly 80% of subjects diagnosed with RLS exhibit 5 PLMs/hour on any single night of polysomnography, and this increases to 90% if two consecutive nights are recorded (52). So, we chose PLMs as an objective index to evaluate the clinical efficacy of acupuncture for RLS. Furthermore, we will assess the subjective and objective sleep and emotional states of RLS patients, as well as the intervention effects of acupuncture.

The pathogenesis of RLS is closely related to the abnormality of the central DA pathway. Neuroimaging technology can improve the specificity and sensitivity of clinical diagnosis of RLS. Functional imaging technologies such as PET-CT and fMRI may help to understand the clinical complexity, intermittentness, and diversity of this disease; improve the specificity and sensitivity of clinical diagnosis; and ultimately provide help for clinical treatment and prognosis. PET uses tracers labeled by radioactive isotopes to study the density of particular receptors or the metabolism and regional cerebral blood flow in specific areas, as well as the different neurotransmitter pathways (53). PET studies consistently support a dysfunction of dopaminergic pathways, involving not only the nigrostriatal pathway but also the mesolimbic pathway (26, 54–58). These studies confirm the dopamine hypothesis of restless legs (59) and are supported by animal research (60). fMRI is an imaging modality based on the blood-oxygen-level-dependent contrast, using hemoglobin as a naturally occurring endogenous contrast agent. It is used to measure brain activity based on the observation that cerebral blood flow and neuronal activation are coupled. Resting-state fMRI is the most-used technique to explore functional connectivity by MRI (61). Neuroimaging studies in RLS indicate a disease-specific dysfunctional cerebral network, involving the basal ganglia, the limbic network, and the sensorimotor system (62). Previous rs-fMRI studies have indicated that patients with RLS may have deficits in controlling and managing sensory information, supporting the hypothesis that RLS could be a disorder of somatosensory processing (54). Iron also plays a significant role in the pathogenesis of RLS (63–65). Currently, central iron monitoring can typically be conducted using Susceptibility Weighted Imaging(SWI) and Quantitative Susceptibility Mapping(QSM) technologies (66–69). SWI and QSM have unique advantages in providing quantitative susceptibility information (70, 71), but they also present higher technical demands and challenges, such as the longer imaging time for QSM, the more complex quantitative analysis methods, and the presence of artifacts in SWI, which can easily affect the results. Therefore, in this study, we chose to use PET-CT to monitor the central dopamine pathway and fMRI to assess brain function following acupuncture intervention in RLS. Through PET-CT and MRI, we will explore whether neuroimaging indicators can improve the specificity and sensitivity of the clinical diagnosis of RLS and further confirm the clinical efficacy of acupuncture in the treatment of RLS. This is the first study to explore the central mechanism of acupuncture for RLS by PET-CT and fMRI. If the results of our study are successful, our findings may help us understand how acupuncture treatment can improve RLS symptoms by affecting dopamine pathways. The dopamine hypothesis of RLS suggests that the condition is related to abnormalities in dopamine signaling in the brain. Furthermore, the sensory system and the limbic system, especially brain areas related to emotional regulation such as the amygdala, also have a close relationship with the dopaminergic pathways. If acupuncture could enhance dopamine activity in the brain by regulating the DAT, this would support the dopamine hypothesis and provide a biological mechanism for the treatment of RLS with acupuncture.

This study will adopt a randomized, single-blind, placebo-controlled clinical trial design to observe the clinical efficacy of acupuncture in the treatment of RLS. We will comprehensively evaluate the clinical efficacy by combining subjective and objective efficacy evaluation tools, which can provide a safe and effective treatment for RLS. The study will further explore the central mechanism of acupuncture treatment of RLS by PET-CT and fMRI. Our research protocol is currently being reported for the first time. If this study successfully demonstrates the efficacy of acupuncture for RLS and reveals its mechanism of action, it may significantly impact the clinical treatment of RLS. Firstly, it may increase the acceptance of acupuncture as a treatment option for RLS, particularly among patients seeking non-pharmacological interventions or those with contraindications to pharmacological treatments. Secondly, understanding how acupuncture works through the dopaminergic pathways can assist physicians in guiding the development of personalized treatment plans. Lastly, the results of this study may stimulate further research to optimize acupuncture treatment protocols and integrate them with other therapeutic approaches, such as pharmacological treatments and lifestyle modifications, thereby providing a more comprehensive management strategy for RLS.
